# Efficacy and safety of bifidobacterium quadruple viable tablets in the treatment of *Helicobacter pylori*-infected peptic ulcer or gastritis patients: a systematic review and meta-analysis

**DOI:** 10.1186/s12879-023-08211-1

**Published:** 2023-05-09

**Authors:** Xueliang Jiang, Chunjin Xu, Bo Liu, Ping Chen, Qinchang Xu, Lu Zhang

**Affiliations:** 1 Digestive Disease Center, Second Affiliated Hospital of Shandong University of Traditional Chinese Medicine, Shandong, 250000 P.R. China; 2 Department of Gastroenterology, First People’s Hospital of Shangqiu City, Henan, 475000 P.R. China; 3 Department of Gastroenterology, First People’s Hospital of the city of Xiangyang, Hubei, 441000 P.R. China; 4 Gastroenterology Department, Chongqing University Three Gorges Hospital, Chongqing, 404100 P. R. China; 5 Hangzhou Grand Biologics Pharmaceutical Co. LTD., Hangzhou, 310000 P. R. China

**Keywords:** Bifidobacterium, Helicobacter pylori, Peptic ulcer, Meta-analysis

## Abstract

**Background:**

To better understand the efficacy and safety of Bifidobacterium quadruple viable tablets in the treatment of *helicobacter pylori* (*H. pylori*)-infected peptic ulcer or gastritis patients.

**Methods:**

A systematic review of the studies published to June 2022 was performed in English database PubMed, Embase, Chinese database CNKI, Wanfang. There were 17 studies were included in this systematic review and meta-analysis. The outcomes measured included *H. pylori* eradication rate, changes in clinical symptoms of epigastric pain scores, and the incidence of adverse reactions.

**Results:**

The results of the fixed effect model showed that the eradication rate of *H. pylori* in the combination of Bifidobacterium quadruple viable bacteria tablets combined with bismuth-containing conventional quadruple therapy was greater than that of bismuth-containing conventional quadruple therapy, and the difference was statistically significant (OR = 3.73, 95%CI (2.79,5.00), Z = 2.78, P < 0.001; I^2^ = 0.0%, P > 0.999). The results of random effects model showed that the epigastric pain score of Bifidobacterium quadruple viable bacteria tablets combined with bismuth-containing conventional quadruple therapy was lower than that of bismuth-containing conventional quadruple therapy, and the difference was statistically significant (WMD=-0.70, 95%CI (-1.06,-0.34), Z = 3.82, P < 0.001; I^2^ = 96.7%, P < 0.001). The results of random effects model showed that the acid reflux score of Bifidobacterium quadruple viable bacteria tablets combined with bismuth-containing conventional quadruple therapy was lower than that of bismuth-containing conventional quadruple therapy, and the difference was statistically significant (WMD=-0.98, 95%CI (-1.70,-0.26), Z = 2.66, P < 0.001; I^2^ = 99.7%, P < 0.001).

**Conclusions:**

The eradication rate of *H. pylori* by Bifidobacterium quadruple viable bacteria tablets combined with bismuth-containing quadruple therapy is better than that of bismuth-containing quadruple therapy. The improvement of clinical symptoms of patients is better than that of bismuth-containing quadruple therapy, and the incidence of adverse reactions is lower than that of bismuth-containing quadruple therapy. Bifidobacterium quadruple viable bacteria tablet combined with bismuth-containing quadruple therapy was effective and safe. It provides a new way to treat patients with *H. pylori*.

## Introduction

*Helicobacter pylori* (*H. pylori*) is the most common pathogen with prevalence rates of more than 80% in developing countries [1]. The cag-PAI was found to be present in more than 96% of 594 isolates in one study [2], which is similar to that reported previously for eastern populations [3–5]. Previous studies reported that cagA and cagE were detected in 66% and 62% *H. pylori* strains respectively in America [6, 7]. The presence of cagA and cagE in *H. pylori* were isolated from Chinese, Indian, and Malay patients in Singapore ranged from 92.3 to 100% [3]. The *H. pylori* was found to be associated to many diseases including chronic gastritis, peptic ulcer disease, gastric mucosa associated lymphoid tissue lymphoma, and gastric adenocarcinoma [8–13]. Antibiotics are most commonly used clinically for the treatment of diseases associated with *H. pylori*. The randomized controlled trial showed that probiotic did not reduce the risk of antibiotic-associated diarrhea in children when analyzed according to the most stringent definition [14]. However, it reduced the overall risk of diarrhea for 7 days after antibiotic treatment. Sheu et al. found that pretreatment of yogurt containing Lactobacillus and Bifidobacterium-containing improved the efficacy of quadruple therapy in eradicating residual *H. pylori* infection after failed triple therapy [15]. There was a study have shown that antibiotics used to treat *H. pylori* may cause microbiome disorders [16]. He et al. found the probiotic could downregulate immune-inflammatory mediators, and modify clinical symptoms in patients [17]. However, there was no significant effect on the eradication rate of *H. pylori*. Francesco et al. found that the treatment seem to improve the eubiosis of the gut microbial consortium [18]. But a study has shown that certain potentially pathogenic bacteria such as Fusobacterium increased after probiotic monotherapy. To better understand the efficacy and safety of Bifidobacterium quadruple viable tablets in the treatment of *H. pylori*-infected peptic ulcer patients. Therefore a meta-analysis was used to measure the effect of Bifidobacterium quadruple viable tablets on patients with *H. pylori*.

## Materials and methods

### Search of literature

We performed this meta-analysis following Preferred Reporting Items for Systematic Reviews and Meta-Analyses (PRISMA) guidelines (https://guides.lib.monash.edu/systematic-review/prisma). PRISMA is an evidence-based minimum set of items for reporting in systematic reviews and meta-analyses. PRISMA is an international initiative developed by relevant experts to address the ongoing issue of a lack of well documented and transparent review methods reported in published review papers. All the prospective or retrospective study published from the database inception through June 2022, were searched from two English-language databases (PubMed and Embase) and two Chinese-language databases (China National Knowledge Infrastructure and Wanfang) by 2 reviewers. Search terms were ((bifidobaeterium tetravaccine tables), AND (bifidobaeterium), AND (*Helicobacter Pylori* OR HP). Quadruple viable tablets: *Bifidobacterium infantis, Feedadditive Lactobacillusacidophilus, Enterococcus faecalis, Bacilluscereus.*

### Inclusion and exclusion criteria

The inclusion criteria included: (1) randomized controlled trials; (2) Patients included in the study were patients with peptic ulcer or gastritis ulcer with *H pylori* infection; (3) The experimental group was Bifidobacterium quadruple viable tablet combined with bismuth-containing quadruple therapy, and the control group was bismuth-containing quadruple therapy; (4) The main indicators are *Helicobacter pylori* clearance rate and symptom score; the secondary indicators are the incidence of adverse reactions.

The exclusion criteria were (1) reduplicative article; (2) conference summaries, comments, letters, etc.; (3) animal studies, existing meta-analyses and systematic reviews; (4) Types of non-randomized controlled trials; (5) No primary indicator data to report.

### Data extraction

Extracted information includes author’s name, publication time, sample size, age of included patients, *H pylori* detection method, outcome indicators, etc. Data were extracted from the literature by the first reviewer, and accuracy was confirmed by the second reviewer.

The risk of bias assessment of the included studies was performed using the Cochrane Collaboration’s RCT risk of bias assessment tool. The Cochrane collaboration risk of the bias tool considers these items for assessment: random sequences generation (for selection bias); allocation concealment (for selection bias); blinding of participants and personnel (for performance bias); blinding of outcome assessment (for detection bias); incomplete outcome data (for attrition bias); selective reporting and other bias (for reporting bias). The two authors performed the risk of bias assessment independently. If there was any disagreement, they discussed with the third author and finally reached agreement.

### Statistical analysis

Data analysis was performed using Stata 15.0 software. The main indicators of *Helicobacter pylori* eradication rate and incidence of adverse events were estimated using indicators OR values ​​and 95%CI. Effects of symptom scores were estimated using Weighted Mean Difference (WMD) and 95% CI. According to the results of the heterogeneity test, choose a random-effects or fixed-effects model to estimate the total effect. Q-test and I^2^-test were used to estimate heterogeneity between studies. When P > 0.1 and I^2^ ≤ 50%, the fixed effect model was used. When P < 0.1 and I^2^ ≥ 50%, the random effect model was used. Funnel plots and Egger’s test were used to assess the primary outcome of publication bias. If the P value is < 0.05, the difference in means is considered statistically significant. Sensitivity analysis was used to identify the sources of heterogeneity, and was performed to cascade studies to observe the effect on the combined effect and the stability of the main index results.

## Results

### Study selection

**Fig. 1:**
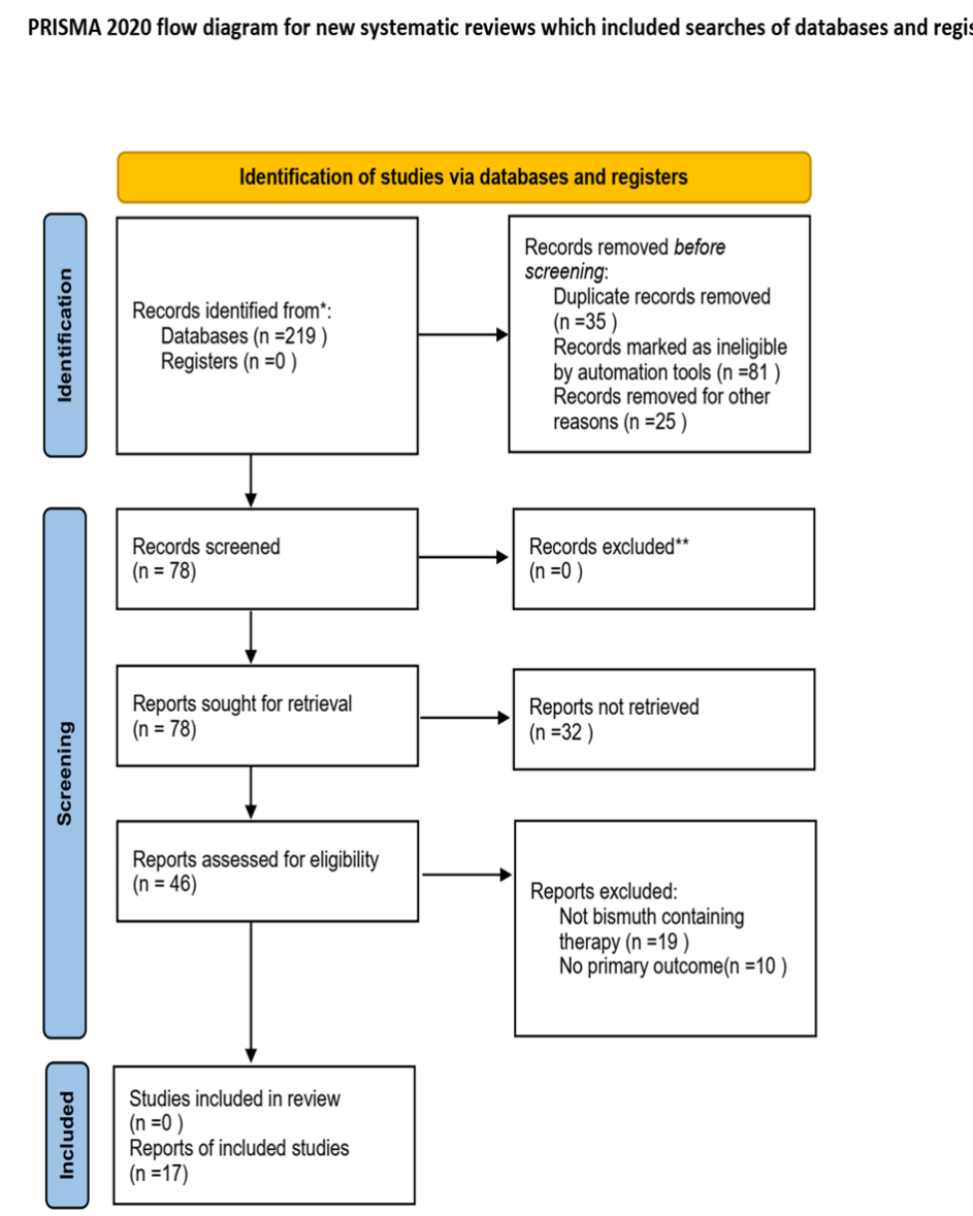
The process of selecting articles for the meta-analysis

**Fig. 2:**
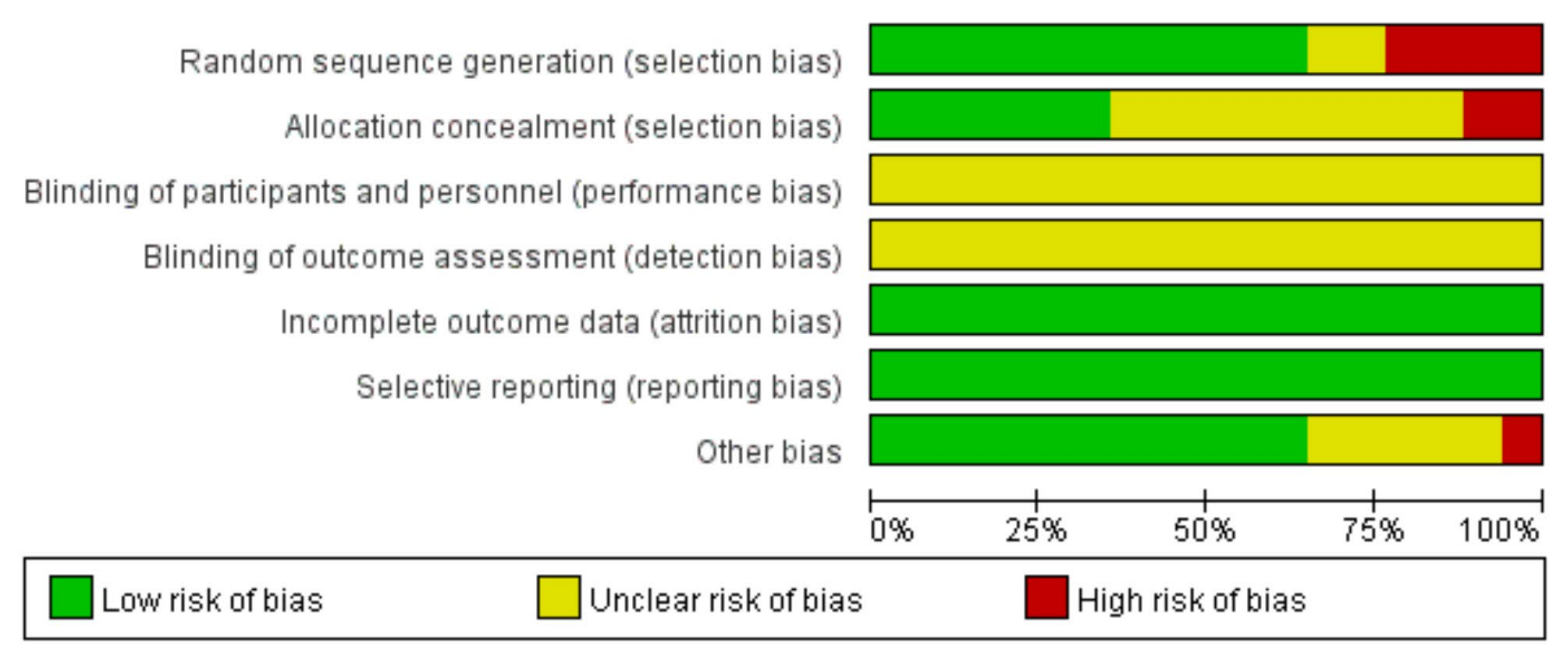
Methodological quality evaluation of included studies

**Fig. 3:**
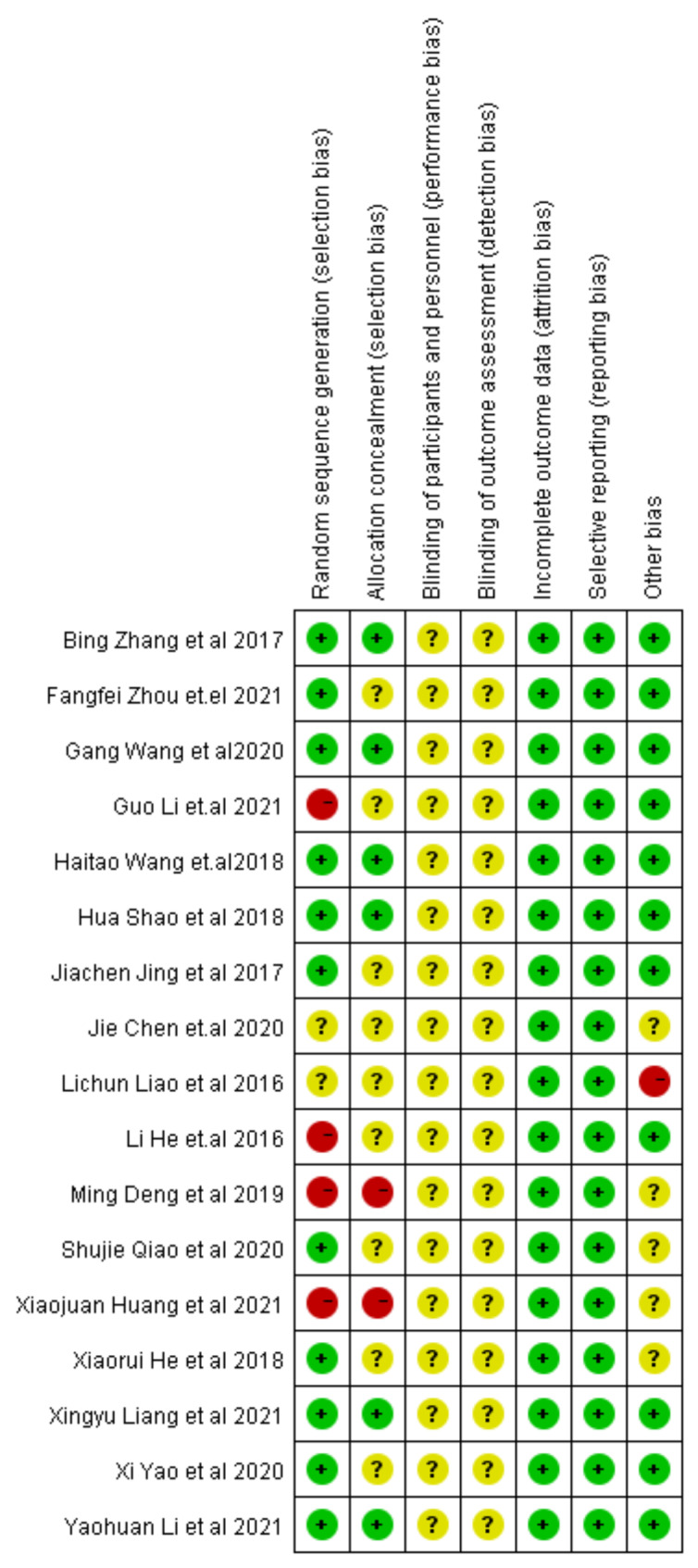
Risk of bias summary

A total of 219 studies were identified through the database search. 35 duplicated reports were excluded. 106 irrelevant studies were excluded after a title and abstract screening that. According to the inclusion and exclusion criteria, 61 studies were excluded, 17 studies met the inclusion criteria. A specific studies flowchart was shown in Fig. 1. And the selected study characteristics were listed in Table 1. The control group was quadruple viable tablets. Bifidobacterium quadruple viable tablets were added to the control group in the treatment group. The results of the risk bias evaluation of the studies were shown in Figs. 2 and 3. The method of randomization was described in 11 studies. 6 articles was used allocation concealment. None of the studies described blinded settings. All articles were unknown risk. All study data were completed. There was no selective reporting bias in any of the 17 articles. The included studies were of good quality and had a low risk of bias.

**Table 1:** Characteristics of Studies Included in the Meta-Analysis.

The first author (year)	Group	Sample size	Age	Intervention	Medicine	Method of detection	Outcomes
Guo Li et al. (2021)	treatment	54	42.37 ± 8.62	control group + Bifidobacterium quadruple viable tablets	1.5 g/once,tid,4 week	--	1,2,3,4
	control	54	43.25 ± 9.28	amoxicillin, clarithromycin, omeprazole enteric-coated tablets, colloidal bismuth pectin	bid, 4 week	--	
Li He et al. (2016)	treatment	54	42.8 ± 9.5	control group + Bifidobacterium quadruple viable tablets	bid, 14d	^14^ C	1
	control	52		omeprazole, amoxicillin, Ofloxacin, colloidal bismuth subcitrate	tid, 14d		
Fangfei Zhou et.el (2021)	treatment	48	47.50 ± 9.17	control group + Bifidobacterium quadruple viable tablets	1.5 g/once, bid, 2week	^14^ C	1,5
	control	48	47.00 ± 9.34	amoxicillin, clarithromycin, lansoprazole, potassium bismuth citrate	bid, 2 week		
Jie Chen et al. (2020)	treatment	35	34.22 ± 6.36	control group + Bifidobacterium quadruple viable tablets	1.5 g/once, tid,4 week	^14^ C	2,3,4,5
	control	35	36.27 ± 4.95	clarithromycin, Ilaprazole enteric-coated tablets, Ornidazole capsules, colloidal bismuth pectin	bid, 4week		
Haitao Wang et al. (2018)	treatment	50	38.1 ± 8.3	control group + Bifidobacterium quadruple viable tablets	1.0 g/once, tid,8 week	^14^ C	1,2,3,4,5
	control	50	37.2 ± 5.1	clarithromycin, Ilaprazole enteric-coated tablets, Ornidazole capsules, colloidal bismuth pectin	8 week		
Hua Shao et al. (2018)	treatment	50	49.39 ± 5.25	control group + Bifidobacterium quadruple viable tablets	1.5 g/once,tid, 2 month	^14^ C	1,5
	control	50	49.03 ± 5.28	amoxicillin, Ilaprazole enteric-coated tablets, Ornidazole capsules, colloidal bismuth pectin	2 month		
Lichun Liao et al. (2016)	treatment	50	40.8 ± 10.4	control group + Bifidobacterium quadruple viable tablets	1.5 g/once,tid, 14 d	^14^ C	1,5
	control	50	44.1 ± 12.7	amoxicillin, omeprazole enteric-coated tablets, Levofloxacin, potassium bismuth citrate	14 day		
Gang Wang et al. (2020)	treatment	56	47.84 ± 11.70	control group + Bifidobacterium quadruple viable tablets	1.4 g/once,bid, 14 d	--	1,5
	control	56	47.64 ± 11.67	amoxicillin, Esomeprazole magnesium, clarithromycin and colloidal bismuth pectin	14 d		
Jiachen Jing et al. (2017)	treatment	80	27–70	control group + Bifidobacterium quadruple viable tablets	tid, 14 d	^13^ C	1
	control	80	28–69	Esomeprazole magnesium, clarithromycin, amoxicillin, colloidal bismuth pectin	14 d		
Xi Yao et al. (2020)	treatment	43	46.5 ± 5.7	control group + Bifidobacterium quadruple viable tablets	tid, 2 month	^14^ C	1
	control	41	46.4 ± 5.6	Esomeprazole magnesium, clarithromycin, Ornidazole capsules, colloidal bismuth pectin	2 month		
Xingyu Liang et al. (2021)	treatment	43	48.27 ± 5.30	control group + Bifidobacterium quadruple viable tablets	tid, 10 d	^14^ C	1,5
	control	43	48.34 ± 5.32	amoxicillin, omeprazole enteric-coated capsule, clarithromycin and colloidal bismuth pectin	10 d		
Ming Deng et al. (2019)	treatment	50	42.06 ± 5.82	control group + Bifidobacterium quadruple viable tablets	4 week	^14^ C	1,3,4,5
	control	50	41.73 ± 6.07	Esomeprazole enteric-soluble capsule, amoxicillin, clarithromycin and colloidal bismuth pectin	4 week		
Xiaojuan Huang et al. (2021)	treatment	57	41.65 ± 8.74	control group + Bifidobacterium quadruple viable tablets	1.5 g/once, bid,2 week	^14^ C	1
	control	57	42.83 ± 9.54	Rabeprazole azole, amoxicillin, clarithromycin and colloidal bismuth pectin	2week		
Yaohuan Li et al. (2021)	treatment	49	35.69 ± 4.22	control group + Bifidobacterium quadruple viable tablets	1.5 g/once, tid,8 week	--	1,5
	control	49	35.65 ± 4.18	amoxicillin, clarithromycin, omeprazole enteric-coated capsule, colloidal bismuth pectin	8week		
Bing Zhang et al. (2017)	treatment	100	58.6 ± 5.1	control group + Bifidobacterium quadruple viable tablets	1.5 g/once,tid,2 week	--	1,5
	control	100	58.4 ± 5.0	potassium bismuth citrate particles, Esomeprazole, Sanzuotong nan, amoxicillin	2 week		
Shujie Qiao et al. (2020)	treatment	70	20 ~ 73	control group + Bifidobacterium quadruple viable tablets	1.5 g/once, bid,2 week	^14^ C	1,5
	control	70	19 ~ 71	Rabeprazole, Stomach bismuth magnesian, amoxicillin, clarithromycin	2 week		
Xiaorui He et al. (2018)	treatment	108		control group + Bifidobacterium quadruple viable tablets	bid,2 week	^14^ C	1
	control	108		amoxicillin, clarithromycin, Lansoprazole tablets, potassium bismuth citrate	2 week		

Note: 1,Hp Eradication Rates; 2, nausea and vomiting; 3, Epigastric pain; 4, acid reflux; 5, adverse reactions; ^14^ C, C14 Breath test; d,day; bid, twice a day; tid, three times a day

**Fig. 4:**
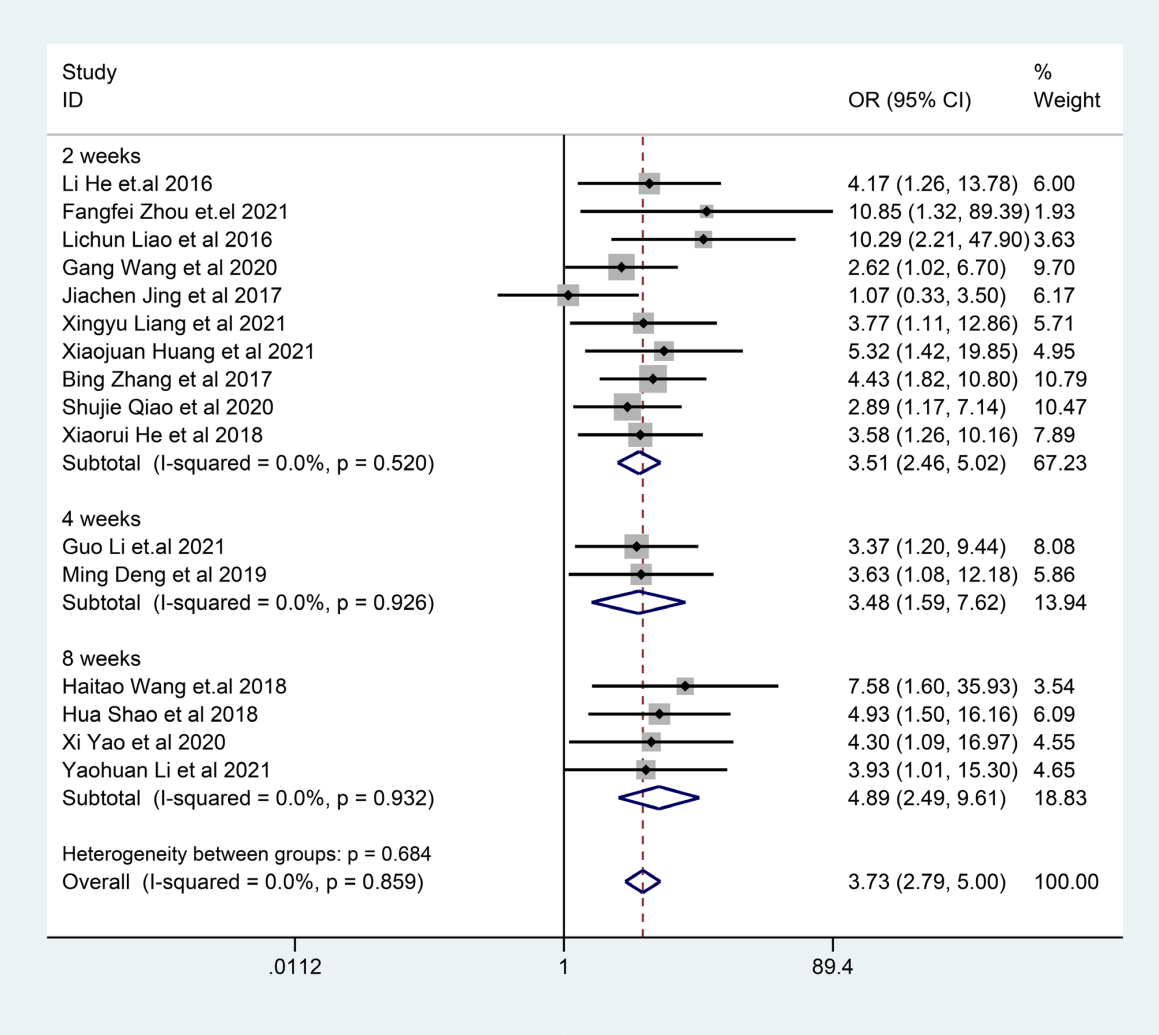
The combined effect results of eradication rate in each study.

### Helicobacter pylori eradication rate

**Fig. 5:**
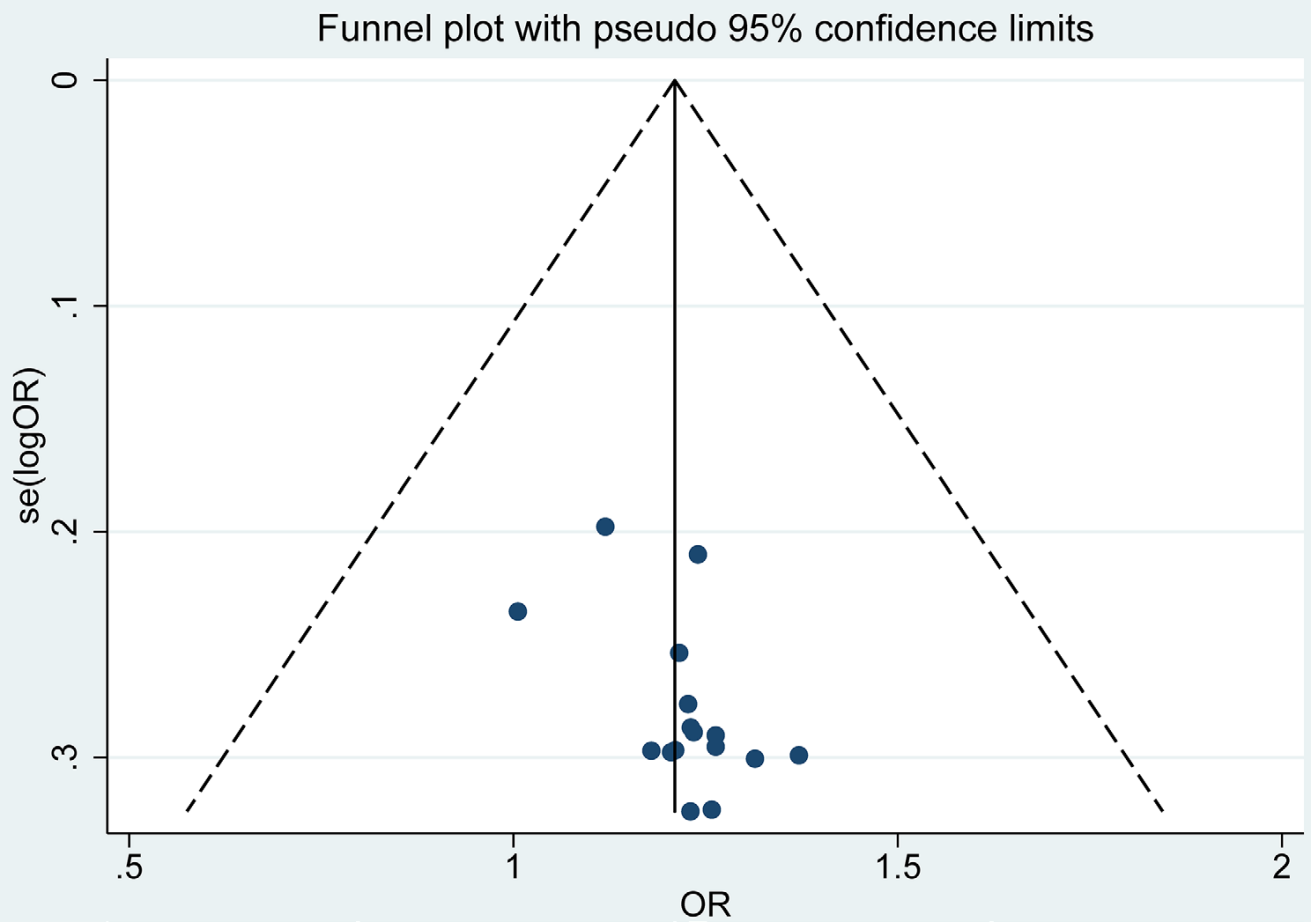
Analysis of publication bias with the funnel plot about the eradication rate

**Fig. 6:**
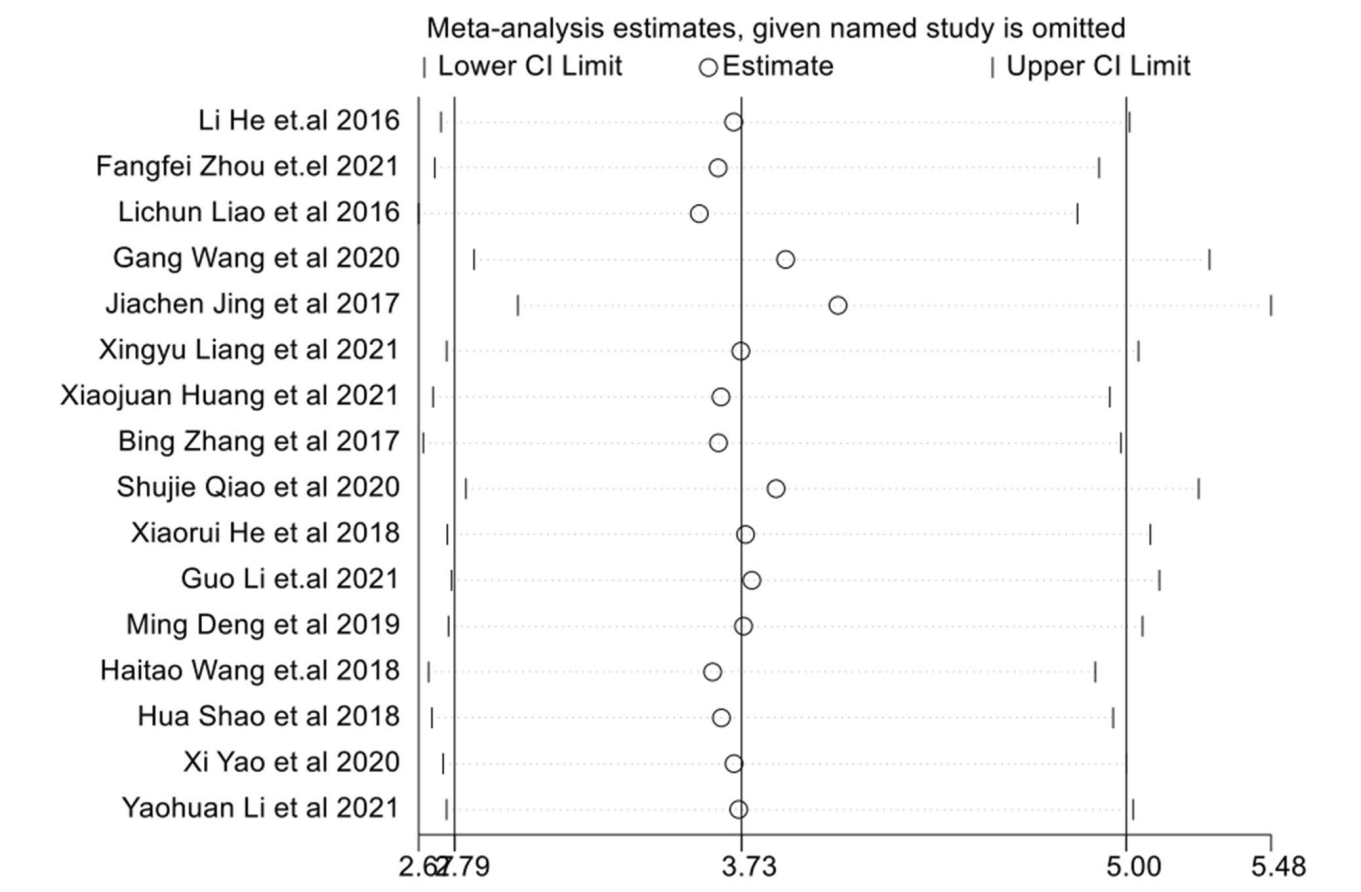
Sensitivity analysis of eradication rate.

The results of the fixed effect model showed that the eradication rate of *H pylori* in the combination of Bifidobacterium quadruple viable bacteria tablets combined with bismuth-containing conventional quadruple therapy was greater than that of bismuth-containing conventional quadruple therapy, and the difference was statistically significant (OR = 3.73, 95%CI (2.79,5.00), Z = 2.78, P < 0.001; I^2^ = 0.0%, P = 0.859). The *H pylori* eradication rate was analyzed by treatment duration as a subgroup (Fig. 4). There was no heterogeneity among studies. The publication bias analysis was shown in Fig. 5. The funnel plot has poor symmetry. Combined with Egger’s test, P < 0.001, there was publication bias. Sensitivity analysis was used to evaluate the stability of the combined effect. By excluding each study step by step, the combined effect was within the 95% CI (1.06, 1.38), and the study results were stable and reliable (Fig. 6).

**Fig. 7:**
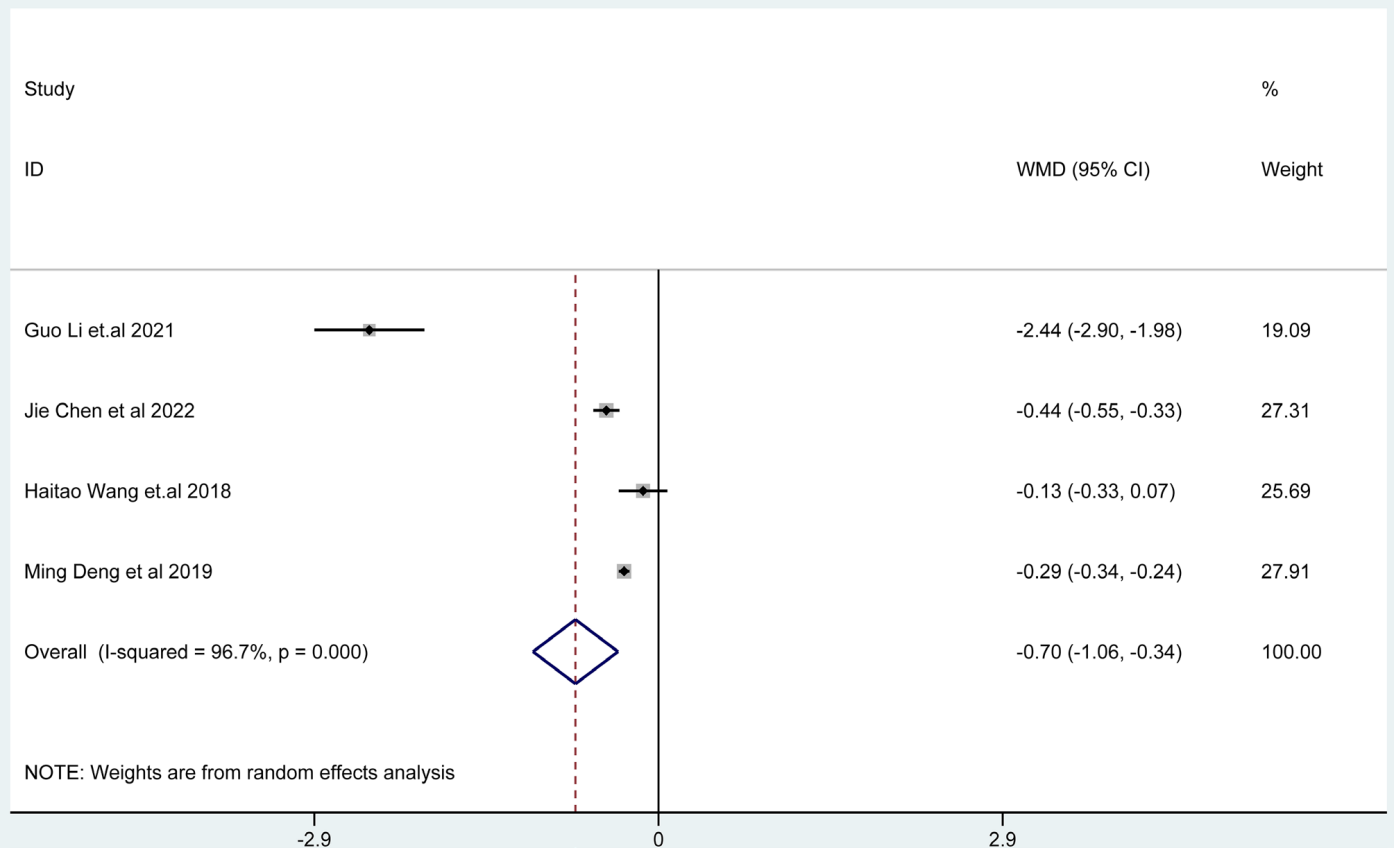
The combined effect results of epigastric pain scores in each study.

### Changes in clinical symptoms of epigastric pain scores

**Fig. 8:**
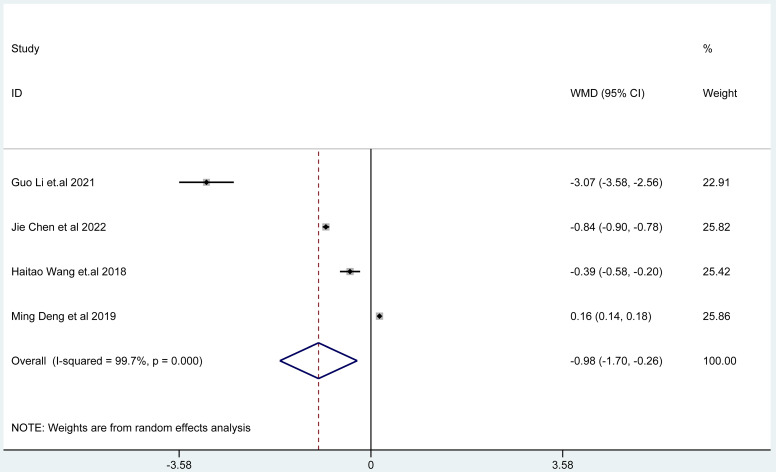
The combined effect results of acid reflux score in each study.

**Fig. 9:**
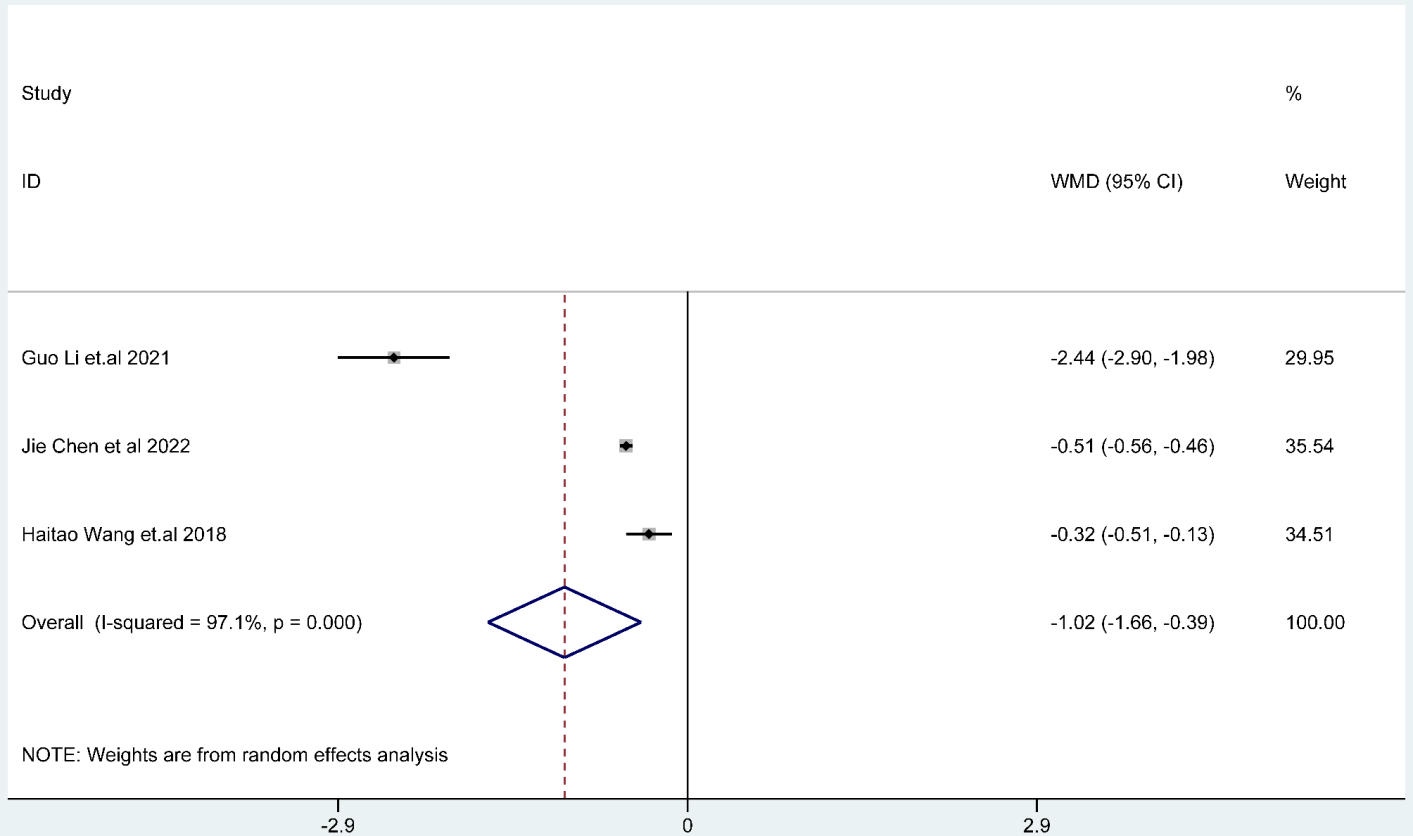
The combined effect results of nausea and vomiting score in each study

The results of random effects model showed that the epigastric pain score of Bifidobacterium quadruple viable bacteria tablets combined with bismuth-containing conventional quadruple therapy was lower than that of bismuth-containing conventional quadruple therapy, and the difference was statistically significant (WMD=-0.70, 95%CI (-1.06,-0.34), Z = 3.82, P < 0.001; I^2^ = 96.7%, P < 0.001) (Fig. 7). There was heterogeneity among the studies. The results of random effects model showed that the acid reflux score of Bifidobacterium quadruple viable bacteria tablets combined with bismuth-containing conventional quadruple therapy was lower than that of bismuth-containing conventional quadruple therapy, and the difference was statistically significant (WMD=-0.98, 95%CI (-1.70,-0.26), Z = 2.66, P < 0.001; I^2^ = 99.7%, P < 0.001) (Fig. 8). There was heterogeneity among the studies. There was heterogeneity among the studies. The results of random effects model showed that the nausea and vomiting score of Bifidobacterium quadruple viable bacteria tablets combined with bismuth-containing conventional quadruple therapy was lower than that of bismuth-containing conventional quadruple therapy, and the difference was statistically significant (WMD=-1.02, 95%CI(-1.66,-0.39), Z = 3.15, P = 0.002; I^2^ = 97.1%, P < 0.001) (Fig. 9). Types of adverse reactions were analyzed in Table 2. There was heterogeneity among the studies.

**Table 2:** Types of adverse reactions were analyzed

Adverse reactions	Pooled effect OR, 95%CI	I^2^,P	Z,P
Loss of appetite	0.57(0.25,1.90)	0.0%,0.839	1.37, P = 0.172
Abdominal distension	0.39(0.16,0.96)	0.0%,0.939	2.05, P = 0.040
Diarrhea	0.30(0.13,0.67)	0.0%,0.526	2.93, P = 0.003
Nausea and vomiting	0.39(0.18,0.82)	0.0%,0.467	2.47, P = 0.014
Nausea	0.37(0.10,1.29)	0.0%,0.891	1.56, P = 0.118
Constipation	0.39(0.19,0.77)	0.0%,0.891	2.68, P = 0.007

### The incidence of adverse reactions

**Fig. 10:**
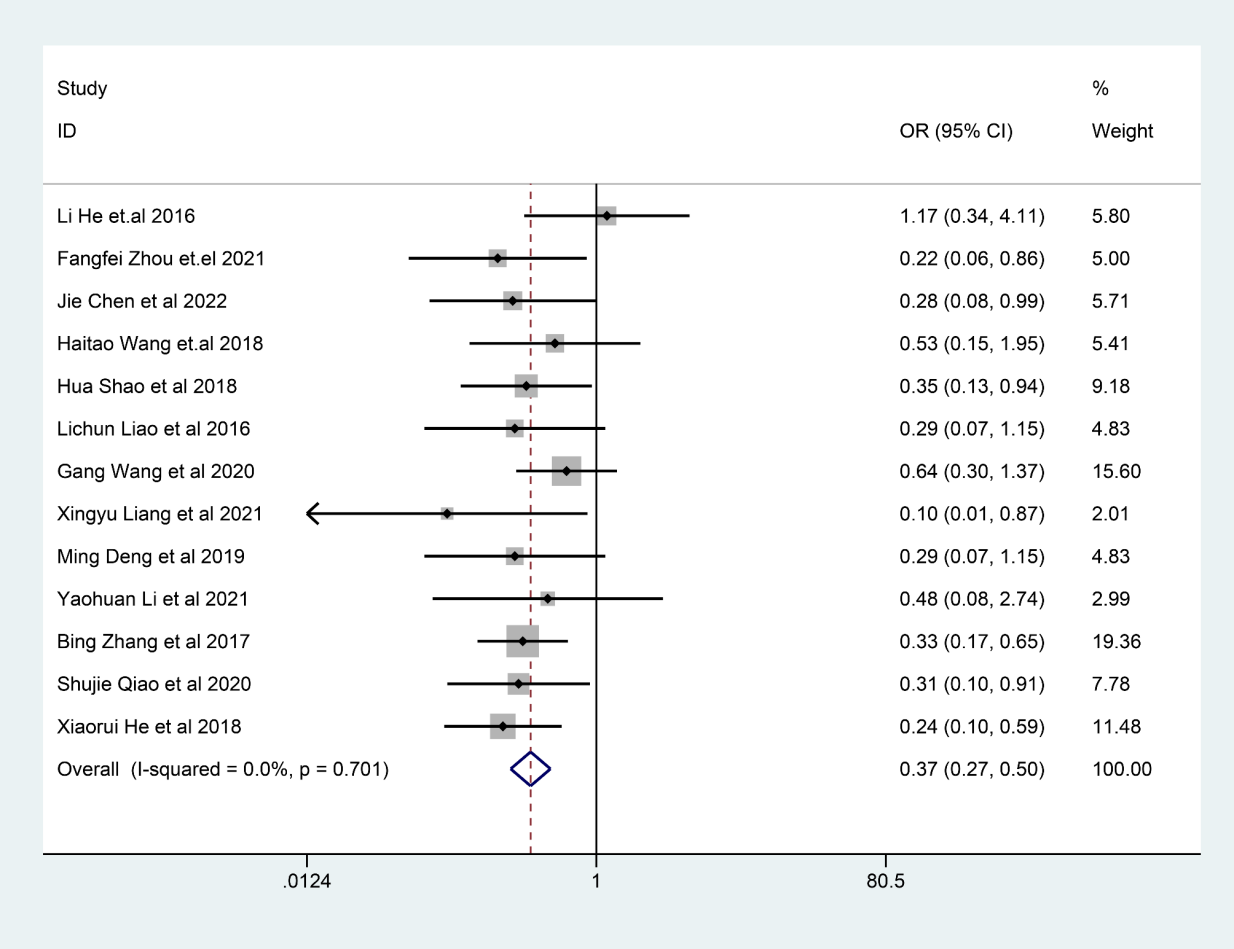
The combined effect results of incidence of adverse reactions in each study

The results of the fixed effect model showed that the incidence of adverse reactions of Bifidobacterium quadruple viable bacteria tablets combined with bismuth-containing conventional quadruple therapy was lower than that of bismuth-containing conventional quadruple therapy, and the difference was statistically significant (OR = 0.37, 95%CI (0.27,0.50), Z = 5.37, P < 0.001; I^2^ = 0.0%, P = 0.782) (Fig. 10). There was no heterogeneity among studies.

## Discussion

The gastric pathogen *H. pylori* is one of the most successful pathogens [19]. *H. pylori* infection is closely related to the pathogenesis of chronic gastritis, peptic ulcer, gastric cancer and gastric mucosa-associated lymphoid tissue lymphoma [20]. The eradication effect of *H. pylori* treatment has decreased owing to increasing its antimicrobial resistance [21]. *H. pylori* eradication therapy includes a variety of drugs, and adverse reactions are common during treatment, especially intestinal flora imbalance [22]. Therefore, many clinical treatment programs use probiotics to regulate the disturbance of intestinal flora while using antibiotics [23]. And some studies have shown that probiotics have an inhibitory effect on the reproduction of HP and can significantly reduce the adverse reactions of drugs and improve the healing rate of gastric mucosal damage [17]. Timely supplementation of probiotics can effectively improve the dysbiosis of the gastrointestinal tract in patients. At the same time, it can effectively protect the gastrointestinal mucosal barrier of patients, so that it can play a better therapeutic effect.

The bifidobacteria in the bifidobacteria quadruple viable tablet can secrete thermostable active protein and have inhibitory effect on Hp. Bifidobacterium is the normal flora of the healthy human gut. Direct supplementation can enhance the biological barrier function of the intestinal mucosa, inhibit the growth and reproduction of pathogenic bacteria, and regulate the intestinal microecological balance. Consistent with our findings, the results of the fixed effect model showed that the eradication rate of *H. pylori* in the combination of Bifidobacterium quadruple viable bacteria tablets combined with bismuth-containing conventional quadruple therapy was greater than that of bismuth-containing conventional quadruple therapy, and the difference was statistically significant. More interestingly, studies have shown that supplementation with yogurt containing bifidobacteria improves *H. pylori* eradication [24].

Fang et al. found Lactobacillus as an adjunct to triple therapy improves *H. pylori* eradication and reduces the incidence of treatment-related diarrhea in children [25]. Results of a meta-analysis of 10 clinical trials showed that lactobacillus- and bifidobacteria-containing probiotic combinations may have a beneficial effect on eradication rates and overall side-effect rates during initial H. pylori eradication therapy in adults [26]. Similarity, the results of random effects model showed that the epigastric pain score of Bifidobacterium quadruple viable bacteria tablets combined with bismuth-containing conventional quadruple therapy was lower than that of bismuth-containing conventional quadruple therapy, and the difference was statistically significant. And we found that the Bifidobacterium quadruple viable bacteria tablets could reduce the incidence of adverse reactions.

## Conclusion

The eradication rate of *H. pylori* by Bifidobacterium quadruple viable bacteria tablets combined with bismuth-containing quadruple therapy was better than that of bismuth-containing quadruple therapy. The improvement of clinical symptoms of patients is better than that of bismuth-containing quadruple therapy, and the incidence of adverse reactions was lower than that of bismuth-containing quadruple therapy. Bifidobacterium quadruple viable bacteria tablet combined with bismuth-containing quadruple therapy was effective and safe.

## Data Availability

Data is obtained with the permission of the corresponding author.
